# Thyrotoxic Periodic Paralysis: An Underdiagnosed and Under-recognized Condition

**DOI:** 10.7759/cureus.342

**Published:** 2015-10-06

**Authors:** Sri Harsha Tella, Anuhya Kommalapati

**Affiliations:** 1 Endocrinology, Diabetes and Metabolism, National Institute of Health; 2 Nutrition, NICHD/NIH; 3 Internal Medicine, NRI Medical College and General Hospital

**Keywords:** thyrotoxicosis, hypokalemia, periodic palsy, graves' disease

## Abstract

Thyrotoxic hypokalemic periodic paralysis (TPP) is a condition characterized by the triad of acute hypokalemia without total body potassium deficit, episodic muscle paralysis, and thyrotoxicosis.

We describe two cases of thyrotoxic periodic paralysis who presented to our hospital with potassium values of 1.3 MeQ/l and 1.2 MeQ/l, respectively. Surprisingly, the two patients had no documented past medical history. Based on the clinical features of high heart rate, palpitations (seen in both the patients), and exophthalmos (seen in one patient), thyrotoxic periodic paralysis was suspected. A thorough laboratory workup confirmed the diagnosis of thyrotoxicosis. Beta blockers were initiated promptly, along with intravenous potassium chloride, and the patients eventually improved symptomatically. These patients were eventually diagnosed with Graves’ disease and were placed on methimazole, which prevented further attacks.

Thyroid periodic paralysis (TPP) is a rare clinical manifestation of hyperthyroidism. Patients present with sudden onset paralysis associated with severe hypokalemia. The presence of paralysis and hypokalemia in a patient who has a history of hyperthyroidism should prompt the physician about thyrotoxic periodic paralysis. A high index of suspicion, prompt diagnosis, and management of the condition can prevent severe complications, such as cardiac arrhythmias.

## Introduction

Thyrotoxic hypokalemic periodic paralysis (TPP) is a condition characterized by the triad of acute hypokalemia without total body potassium deficit, episodic muscle paralysis, and thyrotoxicosis. As hypokalemia has a potential to cause cardiac arrhythmias, early recognition and timely intervention can prevent the mortality in these patients. Intravenous potassium chloride shortens the duration of the attack, but must be given in small quantities and with caution. Non-selective β blockers (eg., Propranolol) play an important role by blocking the Na^+^-K^+^-ATPase pump and, thus, blocking the main pathogenic mechanism in the disease [1-2].

TPP is most commonly found in the East Asian population; however, with globalization and immigration, TPP is no longer limited to certain geographic areas. Although thyrotoxicosis is more common in women, episodes of TPP occur more often in men. We illustrate the characteristics of this condition with the following two case reports in which two Hispanic males presented with acute onset muscle weakness associated with abnormal thyroid function tests.

## Case presentation

Signed informed patient consent was obtained prior to treatment from both patients discussed in this study. No identifying patient information was disclosed in this paper.

### Case presentation 1

A 28-year-old Hispanic male with no significant past medical history presented to our ER with an acute onset weakness of bilateral lower extremities. The patient woke up at 04:45 AM in the morning and was not able to feel his lower extremities, which prompted his wife to call 911. Review of systems was significant for a 10-pound weight loss in three months, prominent eyes, diarrhea (3-6 bowel movements a day), sweating, and palpitations. The patient denied any recent viral infections, ingestion of canned foods, past history or family history of renal disease, sniffing of paint, spinal cord injury, tick bites, and similar episodes of paralysis in the past. The patient was afebrile and had a pulse of 117, RR 14/min, and BP 112/67 mm Hg. On examination, the patient was alert and oriented, had exophthalmos, warm extremities, brisk reflexes, especially in lower extremities, 2/5 motor strength in proximal lower extremities, and 3/5 motor strength in the proximal upper limbs with normal sensation. Laboratory findings were significant for serum potassium - 1.3 MEQ/L (3.5-5.1), free T4 - 5.81 ng/ml (0.60-1.60), TSH - < 0.05 IU/ml (0.34-5.60), free T3 - 9.31 pg/ml (2.5-3.9), magnesium - 1.1 mg/dl (1.2-2.2), calcium - 9.1 mg/dl (8.7-10.6), and phosphorous - 2.0 mg/dl (2.7-4.5). EKG findings were significant for atrial premature complexes and non-specific ST-T changes. The patient was initially managed with 1 mg of propranolol IV along with 40 mg orally every six hours, which was gradually increased to 80 mg every six hours to control symptoms. Potassium was replaced aggressively and cautiously with frequent monitoring of potassium levels (every two hours) to avoid hyperkalemia. Despite close monitoring of potassium levels and cautious replacement, the patient developed rebound hyperkalemia, which was managed symptomatically. The patient’s weakness improved eventually, and his electrolytes were within normal limits. Radioiodine uptake scan showed an increased homogenous uptake of 40% at the end of 24 hours. With the increased thyroid hormone levels in the setting of increased radioiodine uptake, the patient was eventually diagnosed with Graves’ disease. He was discharged on methimazole and propranolol with a scheduled outpatient follow-up. The patient has been compliant with medications and was followed for two years with no recurrence of episodes.

### Case presentation 2

A 26-year-old Hispanic male with no significant past medical history presented to our emergency department as he was not able to move his lower limbs. The patient was apparently asymptomatic the day before. On discussion with the patient, he said that he woke up at 03:00 AM in the morning and felt a little weak in his lower extremities but somehow managed to walk to the restroom and then back to bed. A couple of hours later, he noticed that he was unable to move his legs. The patient denied any recent viral infections, ingestion of canned foods, past history or family history of renal disease, sniffing of paint, spinal cord injury, tick bites, and similar episodes of paralysis in the past. The patient was afebrile and had a pulse of 100, RR 14/min, and a BP 150/67 mm Hg. On examination, the patient was alert and oriented, had sweating, exophthalmos, and warm extremities. Neurological examination was significant for 0/5 motor strength in the proximal lower extremities and 2/5 motor strength in the proximal upper limbs with normal sensation. Laboratory findings were significant for serum potassium - 1.2 MeQ/L (3.5-5.1), magnesium - 0.9 mg/dl (1.2-2.2), calcium - 8.6 mg/dl (8.7-10.6), and phosphorous - 1.9 mg/dl (2.7-4.5). EKG findings were significant for U waves. As the patient was having tachycardia, hypomagnesemia, and hypophosphatemia, thyrotoxicosis periodic paralysis was suspected. His thyroid function tests were as follows: free T4 - 6.57 ng /ml (0.60-1.60), TSH - 0.05 IU/ml (0.34-5.60), and free T3 - 8.78 pg/ml (2.5-3.9). Meanwhile, the patient’s potassium was replaced aggressively with close monitoring of potassium levels. Once thyrotoxicosis was diagnosed, the patient was put on propranolol. The patient improved symptomatically in a couple of days and was sent home on propranolol, 20 mg three times a day. No methimazole was started as the plan was to get a radioactive iodine uptake scan (RAIU) on an outpatient basis. A week later, the patient bounced back to the emergency room with similar complaints. Labs were significant for serum potassium - 1.7 MEQ/L (3.5-5.1), free T4 - 5.97 ng/ml (0.60-1.60), TSH - <0.05 IU/ml (0.34-5.60), free T3 - 9.06 pg/ml (2.5-3.9), magnesium - 1.4 mg/dl (1.2-2.2), calcium - 8.7 mg/dl (8.7-10.6), and phosphorous - 2.4 mg/dl (2.7-4.5). Potassium was replaced and propranolol was started. Once the patient was symptomatically better, thyroid stimulating antibodies were measured, which turned out to be positive (TSI Index of 4.2 (NL: <1.3)). As the patient was started on methimazole, 10 mg thrice a day, we did not get a radioiodine scan. He was followed as an outpatient regularly. Eventually, the patient became euthyroid and no further episodes of paralysis were noted in the 18 months follow-up period.

## Discussion

The present cases describe two Hispanic patients suffering from TPP due to Graves’ disease. Thyrotoxic periodic paralysis (TPP) is a rare life-threatening complication of untreated or unrecognized hyperthyroidism characterized by episodes of muscle weakness and hypokalemia. TPP is much more frequently seen in males in contrast to other forms of thyroid disease, which are more common in women [1]. This diagnosis should be considered when patients present with marked painless weakness associated with hypokalemia and elevated thyroid hormones. Typical signs and symptoms of hyperthyroidism may or may not always be present. TPP occurs in about 0.1 to 0.2% of the hyperthyroid population in North America and usually manifests in the third decade of life as seen in our patients [2]. The association with the presence of HLA-DRW8 suggests that the basic defect may be genetically determined, but the precise pathology of TPP is mainly through Na^+^-K^+^-ATPase pump [3]. The presence of excessive thyroid hormone in serum seems necessary for this disorder. Thyroid hormone, β-adrenergic catecholamine, and insulin can increase the Na^+^-K^+^-ATPase pump activity in skeletal muscles, liver, and kidneys [4]. This leads to a shift of potassium into the cells, manifested as low serum potassium, but without changing the total body potassium level. This may explain why weakness resolves when potassium returns to the extracellular space. Hypophosphatemia and hypomagnesaemia (secondary to the intracellular shift) [5] was also seen in our patients and might have contributed to the muscle weakness [5]. The attacks of paralysis tend to occur during the night, as in our patients, and after stress, alcohol intake, or a carbohydrate-rich meal, suggesting a possible role of hyperinsulinemia [3, 6]. It is also postulated that male hormones increase Na^+^/K^+^-ATPase activity, and that this explains why males are at a higher risk of TPP despite thyroid disease being more common in females [6].

The muscle weakness and increased risk of arrhythmias in TPP result from markedly reduced levels of potassium in serum. An increased Na^+^/K^+^-ATPase activity leads to shifting of potassium into the cells. In other types of potassium derangements, the acid-base balance is usually disturbed, with metabolic alkalosis and metabolic acidosis often being present. In TPP, these disturbances are generally absent. Low potassium levels in the serum lead to hyperpolarization of skeletal muscle cells, making the neuromuscular junction less responsive to normal nerve impulses, which in turn decreases the contractility of the muscles [7].

As with all other periodic paralyses, attacks of weakness occur suddenly with generalized weakness and preserved consciousness. The age of onset of symptoms in TPP is between 20 and 39 years in approximately 80% of patients [8-12]. Although this later age of onset is helpful to distinguish TPP from the familial hypokalemic periodic paralyses, which present at a younger age, adolescents with thyrotoxic periodic paralysis (PP) have been reported [13-14]. In many patients, clinical features of hyperthyroidism precede the onset of PP by months or even years, but they have been noted to occur at the same time (in 43 to 60% of patients) or following the development of PP.

Attacks vary in frequency and duration [9, 15]. Duration of symptoms of several hours is typical, but can range from minutes to days. As with hypokalemic PP, any events that are associated with an increased release of epinephrine or insulin can precipitate TPP, causing movement of potassium into cells and low potassium blood levels [9, 16]. Most commonly, the inciting event is either rest after strenuous physical activity, stress, or a high-carbohydrate load. Other events reported to induce attacks in TPP include cold exposure, infection, alcohol intake, pulse corticosteroid therapy, and menses [9, 12, 17]. In many instances, no obvious precipitant is identified. Although attacks of weakness may occur at any time of the day, a high frequency of attacks at night or early in the morning has been reported in TPP [4].

### Diagnostic evaluation

In an acute attack, TPP must be distinguished from other causes of acute paralysis, such as myasthenic crisis, Guillain-Barré syndrome, acute myelopathy (eg, transverse myelitis), tick paralysis, and botulism [1, 4]. The flaccid paralysis in TPP involves predominantly the muscles of the back, the shoulder, and pelvic girdles. Proximal muscles will be more severely affected than the distal muscles. Myalgia and stiffness are well-recognised complaints. There will be no involvement of the sensory system, and mental function will be generally intact. Usually cranial nerves, bulbar, ocular, and respiratory muscles are spared. Deep tendon reflexes are usually lost, but the anal and bladder sphincter tones are preserved as seen in our patients. These findings differentiate thyrotoxic periodic paralysis from Guillain-Barré syndrome, myasthenia gravis, botulism, and transverse myelitis.

Patients with TPP usually have attacks in the hyperthyroid state. Supporting laboratory findings include elevation of serum thyroxine (T4) and low thyrotropin levels (TSH). Patients with elevated T3 and normal T4 levels have been reported, especially in patients who have an adenoma or Graves’ disease who generally have T3 thyrotoxicosis [18]. Other common laboratory abnormalities include mild hypophosphatemia and hypomagnesemia [4, 12]. These laboratory changes help the clinicians in distinguishing TPP from familial hypokalemic PP [15, 19-20]. Urine calcium to phosphate ratio > 1.7 is a sensitive and specific test to distinguish TPP from familial hypokalemic PP and can be used in case of doubt [21]. Electrocardiogram (EKG) changes are common in an attack of thyrotoxic PP that include the findings consistent with hypokalemia.

### Management

As extracellular potassium is depleted, potassium supplementation leads to improvement of weakness. However, the total body potassium will not change. In this context, one prospective study compared intravenous potassium chloride with normal saline infusion and a shorter recovery time was noted in subjects who received potassium (6.3 versus 13.5 hours) [22]. Similar findings were revealed in a retrospective case series and patients who received intravenous potassium recovered more quickly than those who received oral supplementation [23].

As discussed previously, total body potassium is not altered; hence, rebound hyperkalemia is a prominent problem in thyrotoxic PP, occurring in approximately 40 to 59% of treated attacks [4]. A study has shown that in people who received greater than 90 mEq of potassium in 24 hours, 80% of patients developed rebound hyperkalemia. This can be prevented by giving 30 mEq of oral potassium every two hours until improvement begins, with a maximum dose of 90 mEq in 24 hours [24] or even slower rates were suggested by others (< 10 mEq per hour) [2, 22]. As both high and low serum potassium can cause EKG changes/severe arrhythmias, serial EKGs should be taken; any changes in EKGs should warrant a cardiology consultation. 

Sometimes replacement of potassium may not be sufficient to resolve an attack. As the pathology lies in the Na^+^/K^+^-ATP channel, a beta-adrenergic blocker can presumably reverse the excessive stimulation of the sodium-potassium ATPase and in turn drive the potassium into cells. This is also supported with the fact that intravenous propranolol (1 mg of intravenous propranolol every 10 minutes up to a maximum dose of 3 mg) has been implicated in reversing weakness and hypokalemia in patients with TPP that is unresponsive to potassium administration [17, 26-29].

### Preventive strategies

The main pathology is induced by high thyroxine levels that drive the potassium into cells and so restoration of euthyroidism eliminates attacks of TPP [8]. It has been shown that the administration of beta-blockers, such as propranolol with or without potassium supplementation, has also been shown to decrease the frequency and severity of attacks and should be used until an euthyroid state is achieved [11, 18, 27, 29-31]. Always choose a non-selective beta blocker as β-1 selective agents are less likely to inhibit the β-2 receptor-mediated hypokalemic effect of epinephrine [8]. Carbonic anhydrase inhibitors need a special mention as this class of medications have shown promising benefits in familial hypokalemic periodic paralysis but not in TPP and may even precipitate and increase the attacks [32]. Other precipitating factors, such as heavy exercise, high-carbohydrate diets, and alcohol, should be avoided.

## Conclusions

Patients with TPP can have subtle signs and symptoms of thyrotoxicosis on presentation, and physicians should have a high index of suspicion and a low threshold to get thyroid function tests in these patients who present with acute paralysis associated with hypokalemia. A urine calcium to phosphate ratio > 1.7 is a sensitive and specific test to distinguish TPP from familial hypokalemic PP and can be used when in doubt. Once the diagnosis is confirmed, careful repletion of potassium (total dose of < 90 mEq/24 hours) is recommended with close cardiac monitoring as dysrhythmia risk is high in these patients due to electrolyte imbalances. Prescribing non-selective β-blockers has been shown to be beneficial and should always be considered as it blocks the main pathology in Na^+^-K^+^-ATPase pump. Once the patient is symptomatically improved, a definitive treatment of the underlying thyroid condition has to be considered. TPP needs early recognition for timely intervention, and clinicians should educate the patients to avoid the precipitating factors, such as heavy exercise, high-carbohydrate diets, and alcohol.

## References

[1] Chen YC, Fang JT, Chang CT, Chou HH (2001). Thyrotoxic periodic paralysis in a patient abusing thyroxine for weight reduction. Ren Fail.

[2] Pompeo A, Nepa A, Maddestra M, Feliziani V, Genovesi N (2007). Thyrotoxic hypokalemic periodic paralysis: An overlooked pathology in western countries. Eur J Intern Med.

[3] Hannon MJ, Behan LA, Agha A (2009). Thyrotoxic periodic paralysis due to excessive L-thyroxine replacement in a Caucasian man. Ann Clin Biochem.

[4] Manoukian MA, Foote JA, Crapo LM (1999). Clinical and metabolic features of thyrotoxic periodic paralysis in 24 episodes. Arch Intern Med.

[5] Kung AW (2006). Clinical review: Thyrotoxic periodic paralysis: a diagnostic challenge. J Clin Endocrinol Metab.

[6] Pothiwala P, Levine SN (2010). Analytic review: thyrotoxic periodic paralysis: a review. J Intensive Care Med.

[7] Kung AW (2006). Clinical review: Thyrotoxic periodic paralysis: a diagnostic challenge. J Clin Endocrinol Metab.

[8] Ober KP (1992). Thyrotoxic periodic paralysis in the United States. Report of 7 cases and review of the literature. Medicine (Baltimore).

[9] Hsieh MJ, Lyu RK, Chang WN, Chang KH, Chen CM, Chang HS, Wu YR, Chen ST, Ro LS (2008). Hypokalemic thyrotoxic periodic paralysis: clinical characteristics and predictors of recurrent paralytic attacks. Eur J Neurol.

[10] Okinaka S, Shizume K, Iino S, Watanabe A, Irie M, Noguchi A, Kuma S, Kuma K, Ito T (1957). The association of periodic paralysis and hyperthyroidism in Japan. J Clin Endocrinol Metab.

[11] Rhee EP, Scott JA, Dighe AS (2012). Case records of the Massachusetts General Hospital. Case 4-2012. A 37-year-old man with muscle pain, weakness, and weight loss. N Engl J Med.

[12] Wong P (2003). Hypokalemic thyrotoxic periodic paralysis: a case series. CJEM.

[13] Schalin-Jantti C, Laine T, Valli-Jaakola K, Lonnqvist T, Kontula K, Valimaki MJ (2005). Manifestation, management and molecular analysis of candidate genes in two rare cases of thyrotoxic hypokalemic periodic paralysis. Horm Res.

[14] Wong GW, Leung TF, Lo AF, Ahuja AT, Cheng PS (2000). Thyrotoxic periodic paralysis in a 14-year-old boy. Eur J Pediatr.

[15] Venance SL, Cannon SC, Fialho D, Fontaine B, Hanna MG, Ptacek LJ, Tristani-Firouzi M, Tawil R, Griggs RC, CINCH investigators (2006). The primary periodic paralyses: diagnosis, pathogenesis and treatment. Brain.

[16] Fontaine B, Lapie P, Plassart E, Tabti N, Nicole S, Reboul J, Rime-Davoine CS (1996). Periodic paralysis and voltage-gated ion channels. Kidney Int.

[17] Yu TS, Tseng CF, Chuang YY, Yeung LK, Lu KC (2007). Potassium chloride supplementation alone may not improve hypokalemia in thyrotoxic hypokalemic periodic paralysis. J Emerg Med.

[18] Griggs RC, Bender AN, Tawil R (1996). A puzzling case of periodic paralysis. Muscle Nerve.

[19] Lin SH, Lin YF, Halperin ML (2001). Hypokalaemia and paralysis. QJM.

[20] Hsu YJ, Lin YF, Chau T, Liou JT, Kuo SW, Lin SH (2003). Electrocardiographic manifestations in patients with thyrotoxic periodic paralysis. Am J Med Sci.

[21] Lin SH, Chu P, Cheng CJ, Chu SJ, Hung YJ, Lin YF (2006). Early diagnosis of thyrotoxic periodic paralysis: spot urine calcium to phosphate ratio. Crit Care Med.

[22] Lu KC, Hsu YJ, Chiu JS, Hsu YD, Lin SH (2004). Effects of potassium supplementation on the recovery of thyrotoxic periodic paralysis. Am J Emerg Med.

[23] Cesur M, Bayram F, Temel MA, Ozkaya M, Kocer A, Ertorer ME, Koc F, Kaya A, Gullu S (2008). Thyrotoxic hypokalaemic periodic paralysis in a Turkish population: three new case reports and analysis of the case series. Clin Endocrinol (Oxf).

[24] Abbas MT, Khan FY, Errayes M, Baidaa AD, Haleem AH (2015). Thyrotoxic periodic paralysis admitted to the medical department in Qatar. Neth J Med.

[25] Shiang JC, Cheng CJ, Tsai MK, Hung YJ, Hsu YJ, Yang SS, Chu SJ, Lin SH (2009). Therapeutic analysis in Chinese patients with thyrotoxic periodic paralysis over 6 years. Eur J Endocrinol.

[26] Tassone H, Moulin A, Henderson SO (2004). The pitfalls of potassium replacement in thyrotoxic periodic paralysis: a case report and review of the literature. J Emerg Med.

[27] Shayne P, Hart A (1994). Thyrotoxic periodic paralysis terminated with intravenous propranolol. Ann Emerg Med.

[28] Birkhahn RH, Gaeta TJ, Melniker L (2000). Thyrotoxic periodic paralysis and intravenous propranolol in the emergency setting. J Emerg Med.

[29] Lin SH, Lin YF (2001). Propranolol rapidly reverses paralysis, hypokalemia, and hypophosphatemia in thyrotoxic periodic paralysis. Am J Kidney Dis.

[30] Yeung RT, Tse TF (1974). Thyrotoxic periodic paralysis. Effect of propranolol. Am J Med.

[31] Conway MJ, Seibel JA, Eaton P (1974). Thyrotoxicosis and periodic paralysis: improvement with beta blockade. Ann Intern Med.

[32] Ptácek L (1998). The familial periodic paralyses and nondystrophic myotonias. Am J Med.

